# Absolute Versus Relative Changes in Cardiac Troponins in the Diagnosis of Myocardial Infarction: A Systematic Review and Meta-Analysis

**DOI:** 10.7759/cureus.27414

**Published:** 2022-07-28

**Authors:** Anvesh Ravanavena, Chetna Ravindra, Emmanuelar O Igweonu-Nwakile, Safina Ali, Salomi Paul, Shreyas Yakkali, Sneha Teresa Selvin, Sonu Thomas, Viktoriya Bikeyeva, Ahmed Abdullah, Aleksandra Radivojevic, Anas A Abu Jad, Prachi Balani

**Affiliations:** 1 Internal Medicine, California Institute of Behavioral Neurosciences & Psychology, Fairfield, USA; 2 General Surgery, California Institute of Behavioral Neurosciences & Psychology, Fairfield, USA; 3 Behavioral Neurosciences and Psychology, California Institute of Behavioral Neurosciences & Psychology, Fairfield, USA

**Keywords:** high-sensitivity cardiac troponin (hs-ctn), non-st elevation myocardial infarction, myocardial infarction, delta, relative change, absolute change, cardiac troponin-t, cardiac troponin i

## Abstract

Ischemic heart disease (IHD) is one of the leading causes of death globally. Rapid diagnosis of myocardial infarction (MI) will enable earlier initiation of the treatment and improve patient outcomes. Practice guidelines for non-ST-elevation acute coronary syndromes by the American College of Cardiology (ACC)/American Heart Association (AHA) had listed the diagnostic performance of absolute versus relative changes in evidence gaps. We aimed to address this evidence gap by examining the diagnostic accuracy of absolute versus relative changes in cardiac troponins at various time intervals in diagnosing MI. Grey literature, conference abstracts, animal studies, and reports published before 2009 and in languages other than English were excluded. We included reports that investigated absolute or relative changes in highly sensitive cardiac troponin T (hs-cTnT) or sensitive/highly sensitive cardiac troponin I (s/hs-cTnI) assays after specific time intervals (1, 2, or 3 h) in patients presenting with symptoms suggestive of the acute coronary syndrome. After screening, we arranged the reports in 12 separate groups based on the variables for which the data was reported. Quality assessment of the diagnostic accuracy studies-2 (QUADAS-2) was used to assess the risk of bias in the included studies. The weighted summary area under the curve (AUC) was calculated for each pool. We then performed two-sided (or two-tailed) tests to compare independent receiver operating characteristic (ROC) curves. MedCalc version 20.106 (MedCalc Software Ltd., Ostend, Belgium) was used for all statistical analysis. We included eight reports with 23,450 patients in the meta-analysis.

Weighted summary estimates and their respective 95% confidence intervals (CI) under random-effects model for ROC-AUC are as follows: absolute hs-cTnI at 1 h - 0.94 (95% CI: 0.922 to 0.959, p < 0.001); absolute hs-cTnT at 1 h - 0.921 (95% CI: 0.902 to 0.941, p < 0.001); absolute s/hs-cTnI at 2 h - 0.953 (95% CI: 0.926 to 0.980, p < 0.001); absolute hs-cTnT at 2 h 0.951 (95% CI: 0.940 to 0.962, p < 0.001); relative hs-cTnT at 2 h - 0.818 (95% CI: 0.733 to 0.903, p < 0.001); relative s/hs-cTnI at 2 h - 0.762 (95% CI: 0.726 to 0.798, p < 0.001); absolute hs-cTnI at 3 h - 0.967 (95% CI: 0.95 to 0.984, p < 0.001); absolute hs-cTnT at 3 h - 0.959 (95% CI: 0.950 to 0.968, p < 0.001); and relative hs-cTnT at 3 h - 0.926 (95% CI: 0.907 to 0.945, p < 0.001). P-values of comparison of absolute and relative changes are as follows: hs-cTnT at 1 h: <0.0001; hs-cTnI at 1 h: <0.0001; hs-cTnT at 2 h: 0.0024; s/hs-cTnI at 2 h: <0.0001; hs-cTnT at 3 h: 0.0022; and hs-cTnI at 3 h: 0.0005. Our analysis found absolute changes to be superior to relative changes in both hs-cTnT and s/hs-cTnI at 1, 2, and 3 h in the diagnosis of MI. There was no statistically significant difference in comparing s/hs-cTnI vs. hs-cTnT using absolute or relative changes at any time interval. Our findings suggest that future research investigating a potential 0 h/30 min algorithm should use absolute Δ over relative Δ. A suboptimal number of reports in the groups limited our ability to establish the robustness of the results. We did not receive any funding for this review.

## Introduction and background

Ischemic heart disease (IHD) is one of the leading causes of death globally, with 16% of the world’s total deaths (8.9 million deaths) attributed to it in 2019. From 2000 to 2019, the number of deaths due to IHD increased by more than two million, which was the most significant increase in deaths during this period for any disease [1].

Rapid diagnosis of myocardial infarction (MI) will enable earlier initiation of the treatment and improve patient outcomes. Diagnostic criteria of acute MI are the detection of an increase and/or decrease of cardiac troponin (cTn) values with at least one value above the 99th percentile of the upper reference limit (URL) and at least one of the following: symptoms of myocardial ischemia, new ischemic changes on electrocardiogram (ECG), development of pathological Q waves on ECG, imaging evidence of recent loss of viable myocardium or new abnormality in wall motion in a pattern consistent with an ischemic etiology, and intracoronary thrombus identified on angiography or autopsy. The levels of cTn serve as quantitative markers of myocardial injury. The increase and/or decrease of cTn values represent an acute injury of the myocardium [2]. According to the current universal definition of MI, cardiac troponins are integral to diagnosing acute MI.

The use of cardiac troponin is the most rapidly evolving area in the early diagnosis of the non-ST-elevation acute coronary syndrome [3]. The advent of cardiac troponin T and I assays have seen them outperforming cardiac biomarkers like creatine kinase-myocardial band isoenzyme (CK-MB) and myoglobin, which offered little additional diagnostic value [4]. Technological advancements in sensitive-cardiac troponin (s-cTn) and, later, highly sensitive cardiac troponin assays (hs-cTn assays) had higher sensitivity and diagnostic accuracy. They enabled a more rapid diagnosis of MI than standard cTn assays. The hs-cTn assays have also reduced the diagnosis of unstable angina (UA) and allowed for better differentiation of non-ST-elevation myocardial infarction (NSTEMI) from unstable angina and other cardiac diseases [5]. Serial measurements of hs-cTn assays further increase the diagnostic accuracy. Practice guidelines for the management of patients with non-ST-elevation acute coronary syndromes by the American College of Cardiology (ACC)/American Heart Association (AHA) had listed the diagnostic performance of absolute versus relative changes on serial measurements in evidence gaps [3]. Many studies have investigated absolute and relative changes in cardiac troponins in the diagnosis of MI [6]. This area is relatively less explored in terms of systematic reviews and meta-analyses.

We aimed to address an evidence gap in practice guidelines for a leading global cause of death by examining the diagnostic performance of absolute versus relative changes in cardiac troponins at various time intervals in diagnosing MI.

## Review

Methods

We have followed the guidelines from the Preferred Reporting Items for Systematic Reviews and Meta-Analyses (PRISMA) 2020 statement to conduct and report this systematic review and meta-analysis [7].

Databases and Search Strategies

We used PubMed, PubMed Central (PMC), MEDLINE, and Google Scholar for a comprehensive search to identify the studies. We last searched the above databases in April 2022. Search strategies for various databases are presented in Table 1.

**Table 1:** Search strategies

Databases	Search strategy
PubMed/PMC/MEDLINE	(absolute OR relative OR change* OR delta) AND ("troponin I" OR "Troponin I"[Mesh] OR "troponin T" OR "Troponin T"[Mesh] OR cTnT OR cTnI OR hs-cTnT OR hs-cTnI OR high-sensitivity* OR troponin*) AND (AUC OR "diagnostic accuracy" OR "early diagnos*" OR "Early Diagnosis"[Mesh] OR diagnos* OR "Diagnosis"[Mesh]) AND ("myocardial infarction" OR "Myocardial Infarction"[Mesh] OR MI)
Google Scholar	(absolute relative change delta) AND (troponin) AND (diagnostic accuracy early diagnosis) AND (myocardial infarction)
We explored the reference lists of the retrieved studies for more relevant studies.	

Exclusion and Inclusion Criteria

We screened studies by titles and abstracts based on the following exclusion and inclusion criteria. Animal studies and studies published before 2009 and in languages other than English were filtered out. We excluded grey literature, conference abstracts, and studies investigating cTn changes after cardiac reperfusion. We included studies investigating absolute or relative changes in hs-cTnT or s/hs-cTnI after specific time intervals (1, 2, or 3 h) in patients presenting with symptoms suggestive of acute coronary syndrome. Studies were screened by consensus whenever necessary.

Absolute change and relative change are calculated using the following equations:

$$Absolute\ change\ =\ b-a$$

$$Relative\ change\ =\ (b-a)/a\ \times\ 100$$

$$a\ =\ Result\ of\ the\ baseline\ measurement\ (0\ h)\ at\ admission$$

$$b\ =\ Result\ of\ a\ serial\ measurement\ (at\ 1\ or\ 2\ or\ 3\ h\ after\ admission)$$

Quality Assessment

We used the Quality Assessment of the Diagnostic Accuracy Studies-2 (QUADAS-2) tool to assess the included studies’ quality [8]. A consensus strategy was adopted whenever necessary.

Data Extraction

We initially collected data for our study characteristics table from the included studies. We encountered multiple reports investigating different strategies/hs-cTnI assays by various manufacturers in patients enrolled in the Advantageous Predictors of Acute Coronary Syndromes Evaluation (APACE) study during the same enrolment period. We pooled comparable data from multiple reports from this study only if the patient enrolment period differed. We only pooled data from standalone absolute or relative changes for the s/hs-cTn. We collected the data for the area under the curve (AUC) values and their respective 95% confidence intervals (CI) for all the above variables. Standard error values were then calculated from 95% CI using the following formula: (Upper limit of 95% CI-the lower limit of 95% CI)/3.92 [9].

We did not collect data for metrics like sensitivity, specificity, positive predictive value (PPV), and negative predictive value (NPV). These metrics are reported for a specific cut-off value. Studies have reported data for sensitivity, specificity, PPV, and NPV at different cut-off values of absolute and relative changes. Pooling AUC for receiver operating characteristic (ROC) curves is inherently more reliable in the above context.

Statistical Analysis

We pooled reports separately based on a time interval (1, 2, or 3 h), type of change (absolute or relative), and type of cTn (hs-cTnT or s/hs-cTnI). The weighted summary AUC was then calculated for each pool. Two-sided (or two-tailed) tests were then performed using the weighted summary AUC and standard error (SE) calculated under the random-effects model to test the statistical significance of the difference between the AUC curves of absolute vs. relative changes and hs-cTnT vs. s/hs-cTnI assays. The weighted summary AUC values under the random-effects model of absolute and relative, hs-cTnT and s/hs-cTnT, at 1, 2, and 3 h time intervals were then used to make a visual presentation of AUC trends over time using Microsoft Excel version 2204 (Microsoft Corporation, Redmond, Washington). In cases where there was only one report in a group, we used the AUC value and 95% CI from this single report to test for statistical significance of the difference between the AUC curves and plot the graph depicting AUC trends over time.

We assessed heterogeneity using Cochran’s Q and I^2^ statistics. MedCalc version 20.106 (MedCalc Software Ltd., Ostend, Belgium) was used for all statistical analysis.

Results

**Figure 1:**
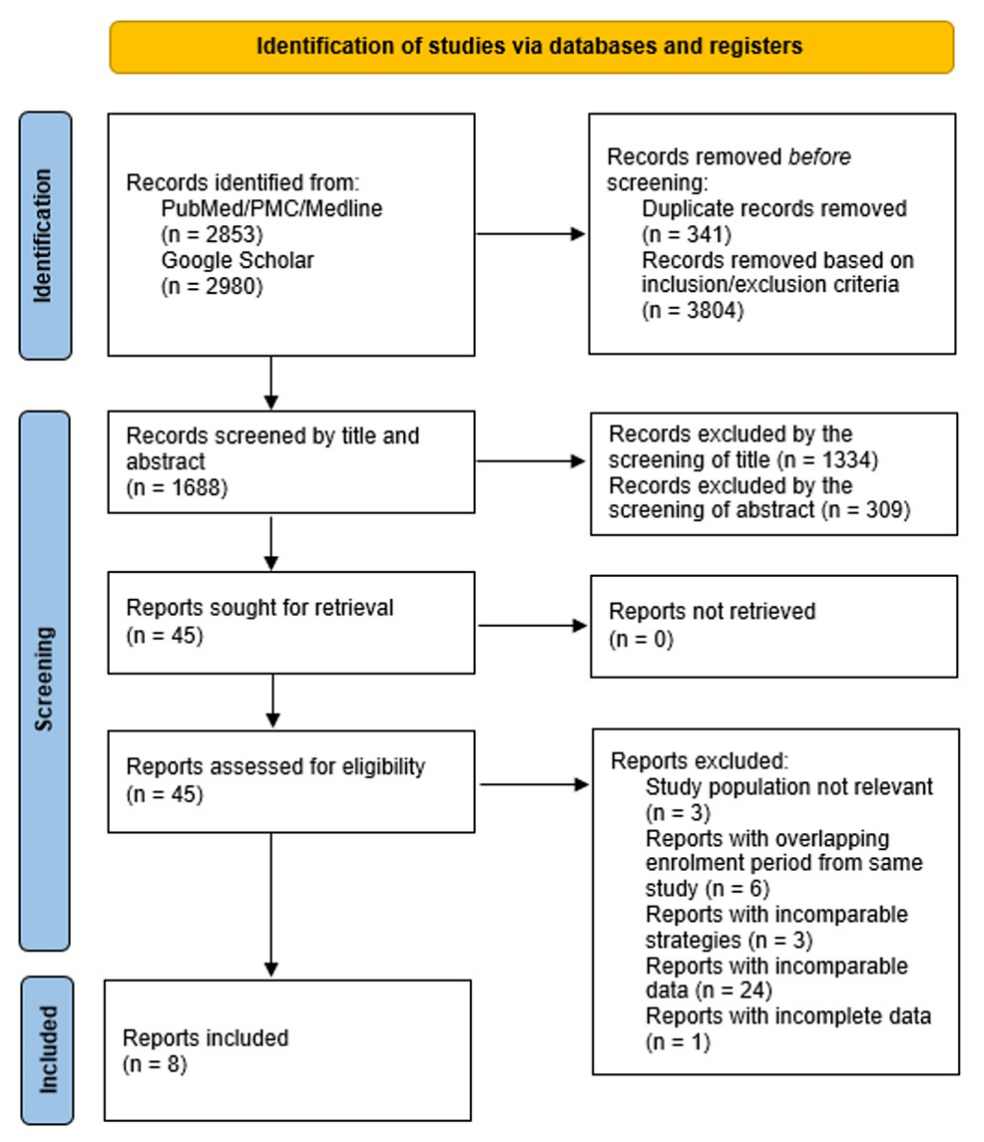
PRISMA (Preferred Reporting Items for Systematic reviews and Meta-Analysis) flow diagram

We sought 45 reports for retrieval, and only eight reports were included in the meta-analysis. Studies reporting absolute or relative changes in combination with variables like baseline cTn > 99th percentile using AND or OR conditions made them incomparable with our data of interest. Some studies did not report AUC values and their respective 95% CI for absolute or relative changes. Many reports investigating different assays and strategies from a single, large, ongoing, international, multicenter study (APACE) were excluded because of overlap in their patient enrolment periods. Reports from this study were grouped only if the enrolment period of the studies did not overlap. The PRISMA flow diagram for identifying studies via databases is presented in Figure 1.

The total number of patients in the eight reports included in the meta-analysis is 23,450. Two out of eight studies included in the meta-analysis are retrospective studies, and all others are prospective studies. Five studies excluded patients with ST-segment elevation myocardial infarction (STEMI). The mean/median age and the percentage of male gender in the total study sample in individual studies ranged from 60 to 67 and 52.7%-78%, respectively, among the reports reporting these data. The mean/median time from symptom onset to the first blood draw in the total study sample in individual studies ranged from 2.75 to 6.3 h, among the reports reporting these data. The characteristics of each included report are presented in Table 2.

**Table 2:** Study characteristics APACE: Advantageous Predictors of Acute Coronary Syndromes Evaluation Study; AMI: Acute myocardial infarction (patients with ST-segment elevation myocardial infarction not excluded in study sample); NSTEMI: Non-ST-segment elevation myocardial infarction; SD: Standard deviation; IQR: Interquartile range; e-GFR: Estimated glomerular filtration rate; hs-cTnT: Highly sensitive cardiac troponin T assay; s-cTnI: Sensitive-cardiac troponin I assay; hs-cTnI: Highly sensitive cardiac troponin I assay; cTnT: Conventional troponin T assay.

Report details	Patients (n)	Study type	Target condition	Age	Male gender (%)	Time from symptom onset to first blood draw	e-GFR/creatinine clearance	Troponin assay(s) used
Aldous et al., 2011 [10]	939	Prospective observational cohort study	NSTEMI	Median 65, IQR (56–76)	59.7	Median 6.3 hours, IQR (3.3–13.3)	Creatinine clearance, mol/L: Median 85, IQR (72–99)	hs-cTnT
Cullen et al., 2013 [11]	874	Prospective diagnostic accuracy study	AMI	AMI: Median 64, IQR (55-81), No AMI: Median 53, IQR (44-63)	60.5	AMI: Median 6.58 hours, IQR (2.3-24.7), No AMI: Median 4.78 hours, IQR (2-18)	Not available	s-cTnI
Irfan et al., 2013 (APACE study) April 2006-June 2009 [12]	830	Prospective, international multicenter study	NSTEMI	Mean 64, SD (51-75)	67	Not available	e-GFR: Mean 89, SD (71-106)	hs-cTnT, hs-cTnI
Kitamura et al., 2013 [13]	85	Prospective, multicenter study	AMI	Median 67, IQR (51–74)	78	Median 165 minutes, IQR (120-180)	e-GFR: Median 76, IQR (60-88)	hs-cTnT, cTnT
Boeddinghaus et al., 2019 (APACE study) June 2010-October 2014 [14]	1579	Prospective, international multicenter study	NSTEMI	Median 60, IQR (48–74)	68	Median 5 hours, IQR (2–12)	Creatinine clearance, mL/min/m^2^: Median 85, IQR (70–101)	hs-cTnI, hs-cTnT
Simpson et al., 2019 [15]	806	Prospective study	NSTEMI	Not available	Not available	Not available	Not available	hs-cTnT
Kim et al., 2020 [16]	281	Retrospective forward observational study	NSTEMI	AMI: 63 (54-77), UA: 67 (59-76), Non-ACS: 55 (46-69) Median (IQR)	66.2	Not available	e-GFR: AMI: 76 (60-87), UA: 77 (59-87), Non-ACS: 83.5 (64-90) Median (IQR)	hs-cTnI
Liu et al., 2022 [17]	18056	Retrospective, observational cohort study	AMI	Mean 63.7, Range (18.1–105.8)	52.7	Not available	e-GFR: Median 75, Range (0.1–328)	hs-cTnT

Reports were placed in 12 separate groups based on the variables for which the data was reported, as shown in Table 3.

**Table 3:** Reports included in various groups for statistical analysis

	Absolute Δ of hs-cTnT	Relative Δ of hs-cTnT	Absolute Δ of s/hs-cTnI	Relative Δ of s/hs-cTnI
1 h	Boeddinghaus et al., 2019 [14], Irfan et al., 2013 [12]	Irfan et al., 2013 [12]	Boeddinghaus et al., 2019 [14], Irfan et al., 2013 [12]	Irfan et al., 2013 [12]
2 h	Boeddinghaus et al., 2019 [14], Irfan et al., 2013 [12], Simpson et al., 2019 [15]	Aldous et al., 2011 [10], Irfan et al., 2013 [12], Simpson et al., 2019 [15]	Boeddinghaus et al., 2019 [14], Cullen et al., 2013 [11], Irfan et al., 2013 [12]	Cullen et al., 2013 [11], Irfan et al., 2013 [12]
3 h	Boeddinghaus et al., 2019 [14], Kitamura et al., 2013 [13], Liu et al., 2022 [17], Simpson et al., 2019 [15]	Kitamura et al., 2013 [13], Simpson et al., 2019 [15]	Boeddinghaus et al., 2019 [14], Kim et al., 2020 [16]	Kim et al., 2020 [16]

Meta-analysis

**Figure 2:**
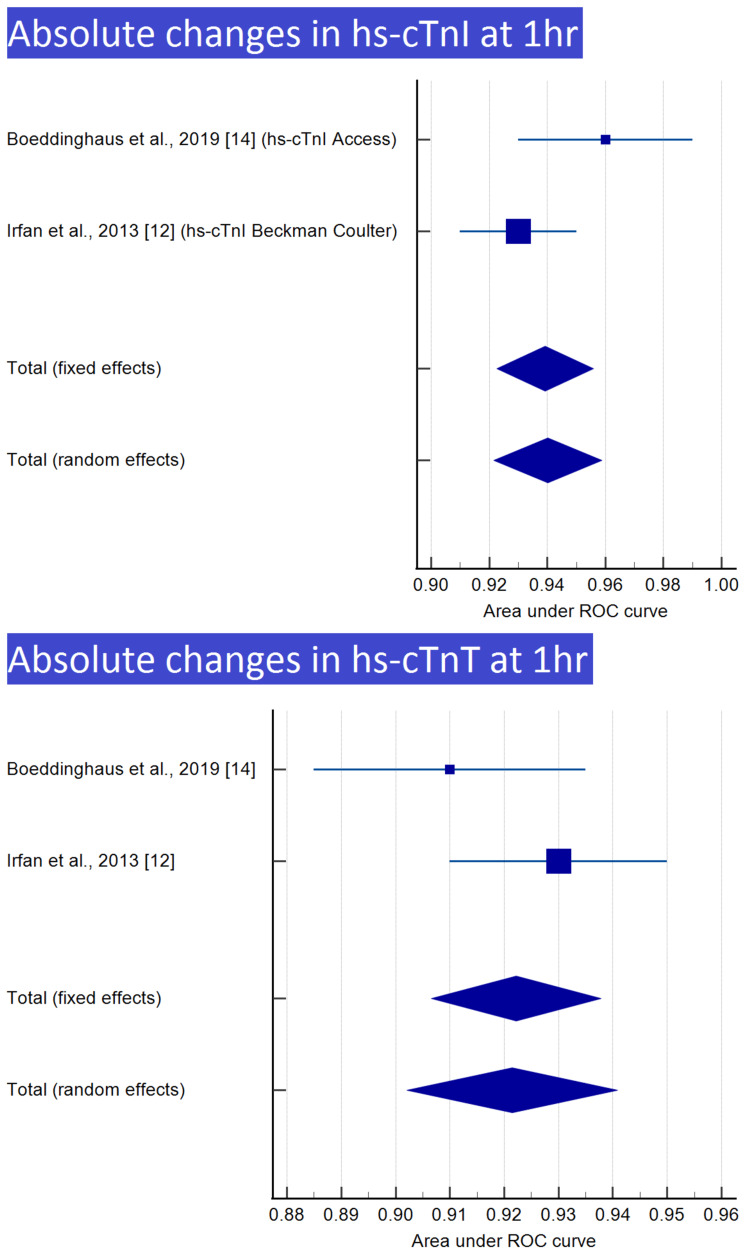
Absolute changes in hs-cTnI and hs-cTnT at 1 h Image credit: Authors of this study.

**Figure 3:**
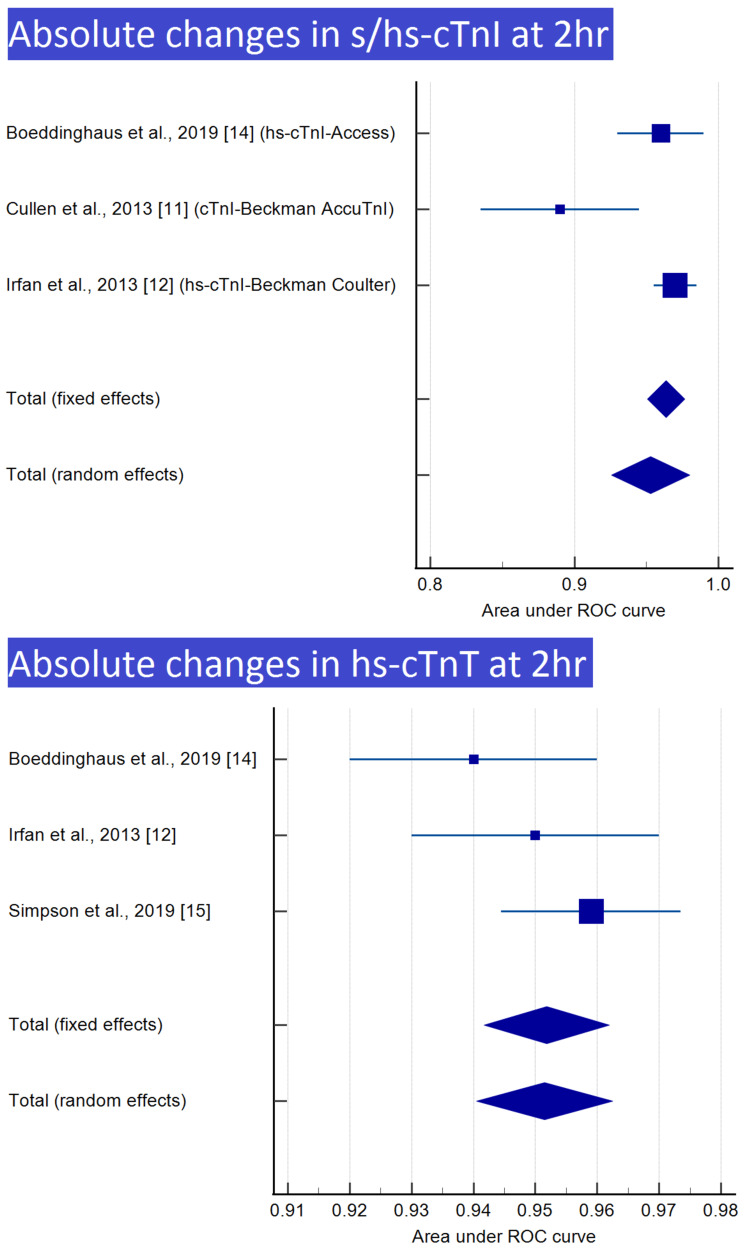
Absolute changes in s/hs-cTnI and hs-cTnT at 2 h Image credit: Authors of this study.

**Figure 4:**
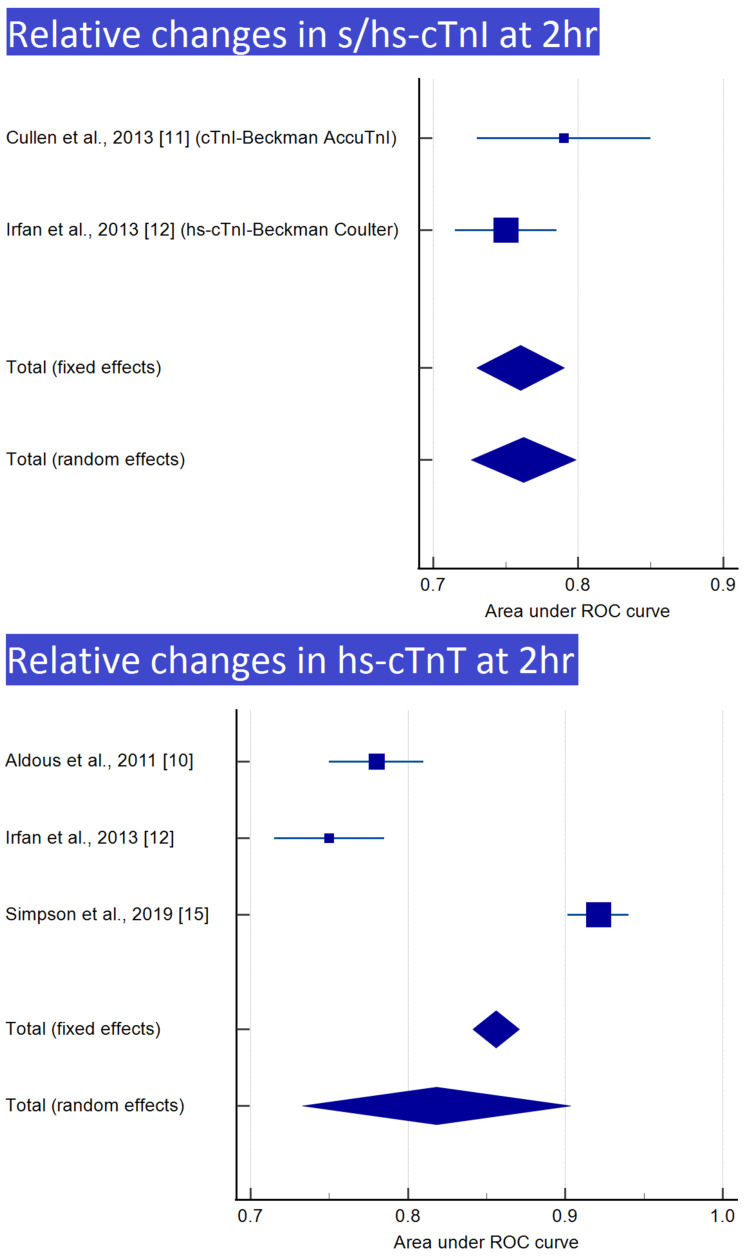
Relative changes in s/hs-cTnI and hs-cTnT at 2 h Image credit: Authors of this study.

**Figure 5:**
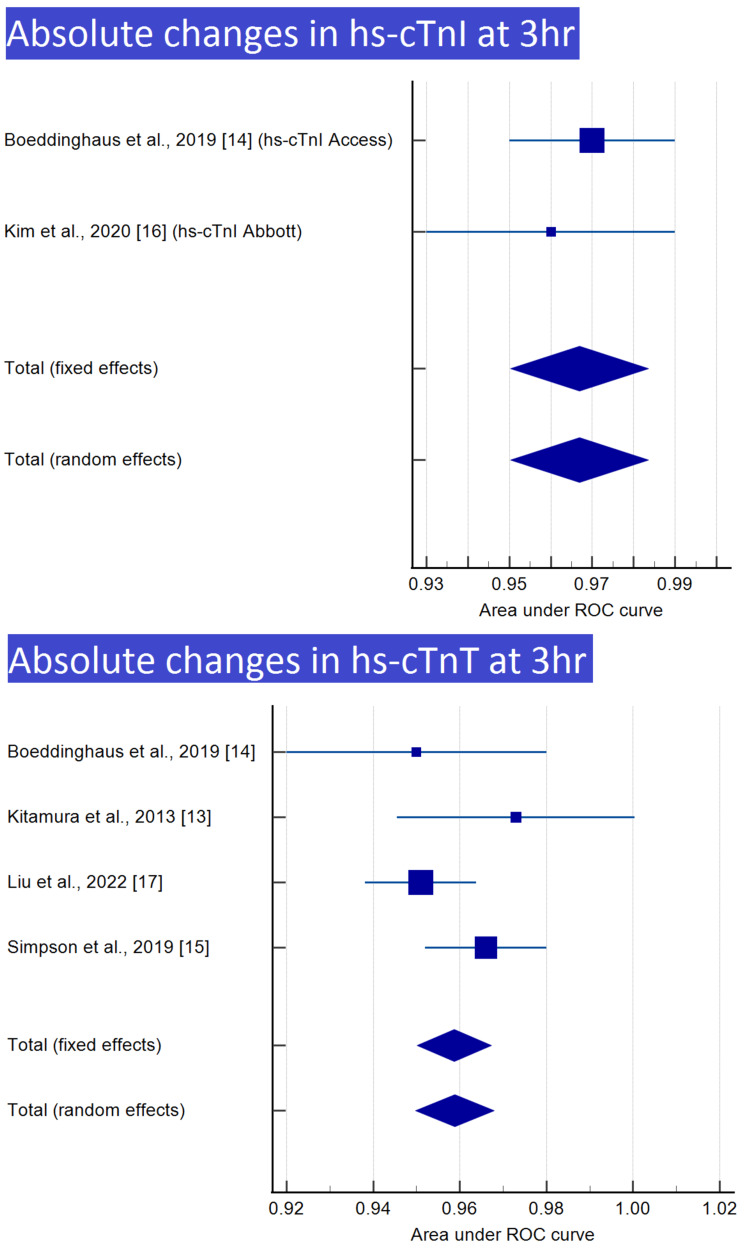
Absolute changes in hs-cTnI and hs-cTnT at 3 h Image credit: Authors of this study.

**Figure 6:**
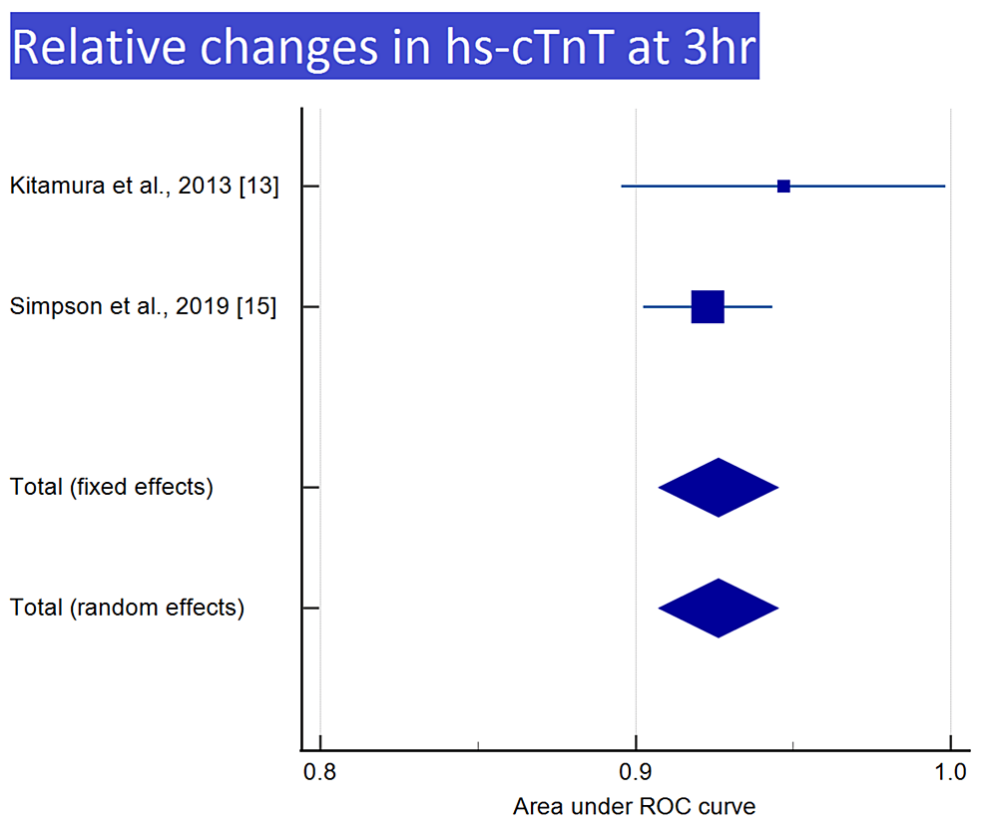
Relative changes in hs-cTnT at 3 h Image credit: Authors of this study.

Meta-analysis was performed for nine of the 12 groups, with at least two reports, as shown in Table 3. Meta-analyses and forest plots of those nine groups are presented in Tables 4-12 and Figures 2-6.

**Table 4:** Absolute changes in hs-cTnI at 1 h

Study	ROC area	Standard error	95% CI	z	P	Weight (%)	
						Fixed	Random
Boeddinghaus et al., 2019 [14] (hs-cTnI access)	0.960	0.0153	0.930 to 0.990			30.77	33.86
Irfan et al., 2013 [12] (hs-cTnI-Beckman Coulter)	0.930	0.0102	0.910 to 0.950			69.23	66.14
Total (fixed effects)	0.939	0.00849	0.923 to 0.956	110.624	<0.001	100.00	100.00
Total (random effects)	0.940	0.00950	0.922 to 0.959	98.956	<0.001	100.00	100.00

**Table 5:** Absolute changes in hs-cTnT at 1 h

Study	ROC area	Standard error	95% CI	z	P	Weight (%)	
						Fixed	Random
Boeddinghaus et al., 2019 [14]	0.910	0.0128	0.885 to 0.935			39.02	42.68
Irfan et al., 2013 [12]	0.930	0.0102	0.910 to 0.950			60.98	57.32
Total (fixed effects)	0.922	0.00797	0.907 to 0.938	115.737	<0.001	100.00	100.00
Total (random effects)	0.921	0.00989	0.902 to 0.941	93.150	<0.001	100.00	100.00

**Table 6:** Absolute changes in s/hs-cTnI at 2 h

Study	ROC area	Standard error	95% CI	z	P	Weight (%)	
						Fixed	Random
Boeddinghaus et al., 2019 [14] (hs-cTnI-access)	0.960	0.0153	0.930 to 0.990			18.88	33.85
Cullen et al., 2013 [11] (cTnI-Beckman AccuTnI)	0.890	0.0281	0.835 to 0.945			5.62	17.17
Irfan et al., 2013 [12] (hs-cTnI-Beckman Coulter)	0.970	0.00765	0.955 to 0.985			75.51	48.98
Total (fixed effects)	0.964	0.00665	0.951 to 0.977	144.902	<0.001	100.00	100.00
Total (random effects)	0.953	0.0139	0.926 to 0.980	68.659	<0.001	100.00	100.00

**Table 7:** Absolute changes in hs-cTnT at 2 h

Study	ROC area	Standard error	95% CI	z	P	Weight (%)	
						Fixed	Random
Boeddinghaus et al., 2019 [14]	0.940	0.0102	0.920 to 0.960			25.62	26.84
Irfan et al., 2013 [12]	0.950	0.0102	0.930 to 0.970			25.62	26.84
Simpson et al., 2019 [15]	0.959	0.00740	0.944 to 0.974			48.75	46.32
Total (fixed effects)	0.952	0.00517	0.942 to 0.962	184.269	<0.001	100.00	100.00
Total (random effects)	0.951	0.00562	0.940 to 0.962	169.451	<0.001	100.00	100.00

**Table 8:** Relative changes in s/hs-cTnI at 2 h

Study	ROC area	Standard error	95% CI	z	P	Weight (%)	
						Fixed	Random
Cullen et al., 2013 [11] (cTnI-Beckman AccuTnI)	0.790	0.0306	0.730 to 0.850			25.39	30.68
Irfan et al., 2013 [12] (hs-cTnI-Beckman Coulter)	0.750	0.0179	0.715 to 0.785			74.61	69.32
Total (fixed effects)	0.760	0.0154	0.730 to 0.790	49.282	<0.001	100.00	100.00
Total (random effects)	0.762	0.0184	0.726 to 0.798	41.323	<0.001	100.00	100.00

**Table 9:** Relative changes in hs-cTnT at 2 h

Study	ROC area	Standard error	95% CI	z	P	Weight (%)	
						Fixed	Random
Aldous et al., 2011 [10]	0.780	0.0153	0.750 to 0.810			24.38	33.23
Irfan et al., 2013 [12]	0.750	0.0179	0.715 to 0.785			17.91	32.74
Simpson et al., 2019 [15]	0.921	0.00995	0.901 to 0.941			57.71	34.04
Total (fixed effects)	0.856	0.00756	0.841 to 0.871	113.261	<0.001	100.00	100.00
Total (random effects)	0.818	0.0434	0.733 to 0.903	18.850	<0.001	100.00	100.00

**Table 10:** Absolute changes in hs-cTnI at 3 h

Study	ROC area	Standard error	95% CI	z	P	Weight (%)	
						Fixed	Random
Boeddinghaus et al., 2019 [14] (hs-cTnI access)	0.970	0.0102	0.950 to 0.990			69.23	69.23
Kim et al., 2020 [16] (hs-cTnI Abbott)	0.960	0.0153	0.930 to 0.990			30.77	30.77
Total (fixed effects)	0.967	0.00849	0.950 to 0.984	113.885	<0.001	100.00	100.00
Total (random effects)	0.967	0.00849	0.950 to 0.984	113.885	<0.001	100.00	100.00

**Table 11:** Absolute changes in hs-cTnT at 3 h

Study	ROC area	Standard error	95% CI	z	P	Weight (%)	
						Fixed	Random
Boeddinghaus et al., 2019 [14]	0.950	0.0153	0.920 to 0.980			8.18	8.92
Kitamura et al., 2013 [13]	0.973	0.0140	0.946 to 1.000			9.77	10.59
Liu et al., 2022 [17]	0.951	0.00656	0.938 to 0.964			44.49	43.22
Simpson et al., 2019 [15]	0.966	0.00714	0.952 to 0.980			37.56	37.26
Total (fixed effects)	0.959	0.00438	0.950 to 0.967	219.095	<0.001	100.00	100.00
Total (random effects)	0.959	0.00463	0.950 to 0.968	206.943	<0.001	100.00	100.00

**Table 12:** Relative changes in hs-cTnT at 3 h

Study	ROC area	Standard error	95% CI	z	P	Weight (%)	
						Fixed	Random
Kitamura et al., 2013 [13]	0.947	0.0263	0.896 to 0.998			13.68	13.68
Simpson et al., 2019 [15]	0.923	0.0105	0.902 to 0.944			86.32	86.32
Total (fixed effects)	0.926	0.00972	0.907 to 0.945	95.320	<0.001	100.00	100.00
Total (random effects)	0.926	0.00972	0.907 to 0.945	95.320	<0.001	100.00	100.00

**Figure 7:**
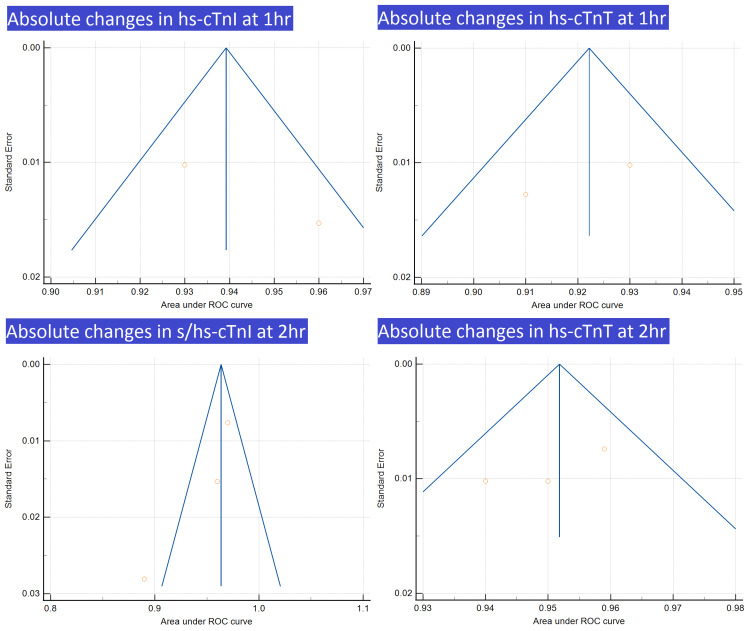
Funnel plots for absolute changes in hs-cTnI at 1 h, absolute changes in hs-cTnT at 1 h, absolute changes in s/hs-cTnI at 2 h, and absolute changes in hs-cTnT at 2 h Image credit: Authors of this study.

**Figure 8:**
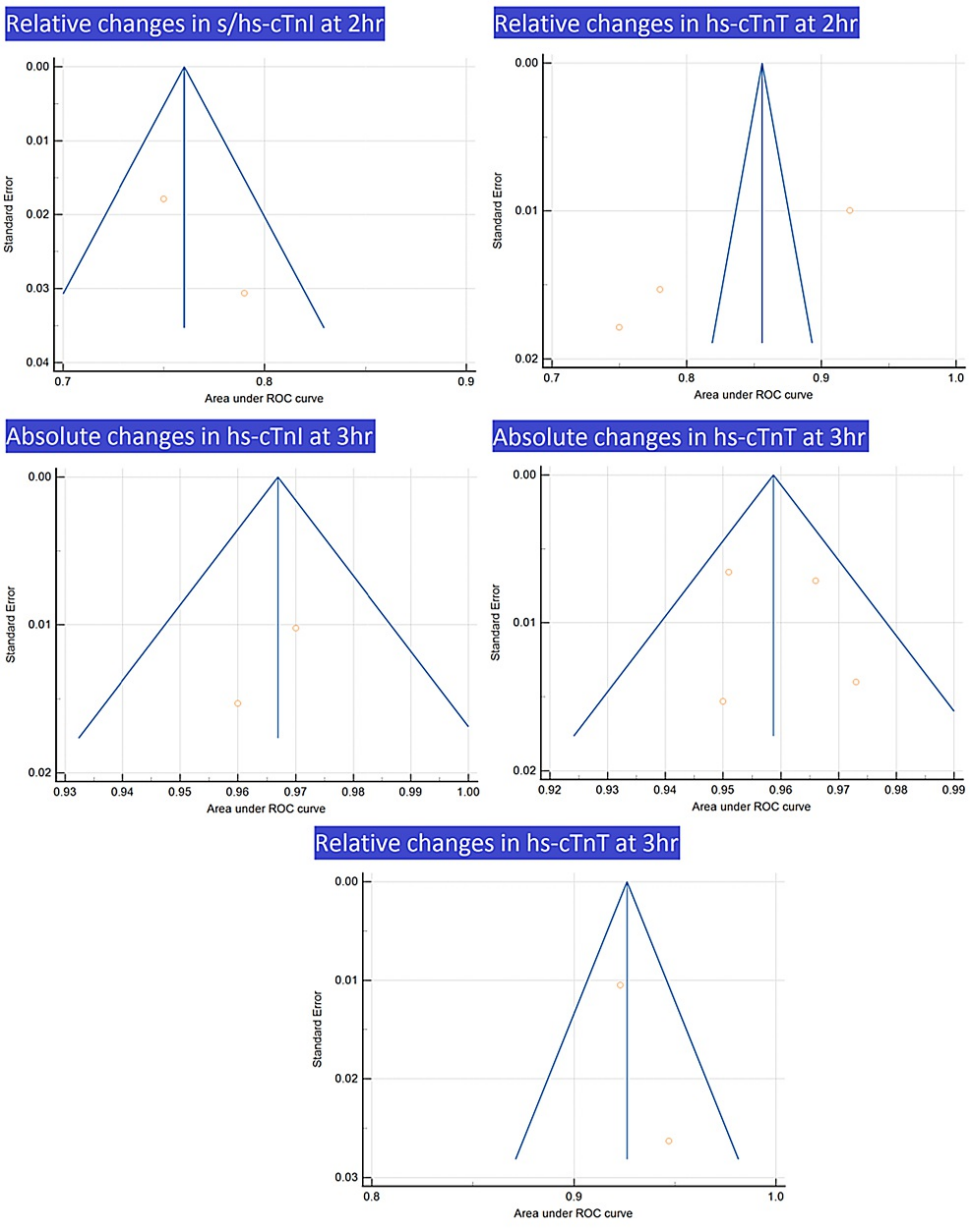
Funnel plots for relative changes in s/hs-cTnI at 2 h, relative changes in hs-cTnT at 2 h, absolute changes in hs-cTnI at 3 h, absolute changes in hs-cTnT at 3 h, and relative changes in hs-cTnT at 3 h Image credit: Authors of this study.

Funnel plots are presented in Figures 7, 8.

Tests for heterogeneity are reported in Table 13 for each meta-analysis performed. They were statistically significant in two groups, as shown in Table 13: Absolute changes in s/hs-cTnI at 2 h and relative changes in hs-cTnT at 2 h.

**Table 13:** Tests for heterogeneity

Meta-analysis	Cochran’s Q	DF	Significance level	I^2^ (inconsistency)	95% CI for I^2^
Absolute changes in hs-cTnI at 1 h	2.6596	1	P = 0.1029	62.40%	0.00 to 91.33
Absolute changes in hs-cTnT at 1 h	1.4992	1	P = 0.2208	33.30%	0.00 to 0.00
Absolute changes in s/hs-cTnI at 2 h	7.6339	2	P = 0.0220	73.80%	12.42 to 92.16
Absolute changes in hs-cTnT at 2 h	2.3155	2	P = 0.3142	13.63%	0.00 to 97.10
Relative changes in s/hs-cTnI at 2 h	1.2739	1	P = 0.2590	21.50%	0.00 to 0.00
Relative changes in hs-cTnT at 2 h	102.5751	2	P < 0.0001	98.05%	96.40 to 98.94
Absolute changes in hs-cTnI at 3 h	0.2955	1	P = 0.5867	0.00%	0.00 to 0.00
Absolute changes in hs-cTnT at 3 h	3.7897	3	P = 0.2851	20.84%	0.00 to 89.78
Relative changes in hs-cTnT at 3 h	0.7202	1	P = 0.3961	0.00%	0.00 to 0.00

Weighted summary estimates of AUC values under the random-effects model are tabulated in Table 14. In groups with only one report, the data from that single report are used.

**Table 14:** AUC values in various groups

	Absolute Δ of hs-cTnT	Relative Δ of hs-cTnT	Absolute Δ of s/hs-cTnI	Relative Δ of s/hs-cTnI
1 h	0.921	0.67	0.94	0.65
2 h	0.951	0.818	0.953	0.762
3 h	0.959	0.926	0.967	0.89

The AUC values in Table 14 and their respective standard errors under the random-effects model in Tables 4-12 and underlying studies (in cases where there is only one report in the group) are used to compare independent ROC curves as shown in Tables 15, 16.

**Table 15:** Comparison of absolute vs. relative changes

Comparison	P-value
Absolute vs relative changes in hs-cTnT at 1 h	<0.0001
Absolute vs relative changes in hs-cTnI at 1 h	<0.0001
Absolute vs relative changes in hs-cTnT at 2 h	0.0024
Absolute vs relative changes in s/hs-cTnI at 2 h	<0.0001
Absolute vs relative changes in hs-cTnT at 3 h	0.0022
Absolute vs relative changes in hs-cTnI at 3 h	0.0005

**Table 16:** Comparison of hs-cTnT vs. s/hs-cTnI

Comparison of hs-cTnT vs. s/hs-cTnI	P-value
Absolute changes at 1 h	0.1659
Relative changes at 1 h	0.3555
Absolute changes at 2 h	0.8939
Relative changes at 2 h	0.2348
Absolute changes at 3 h	0.4081
Relative changes at 3 h	0.1113

**Figure 9:**
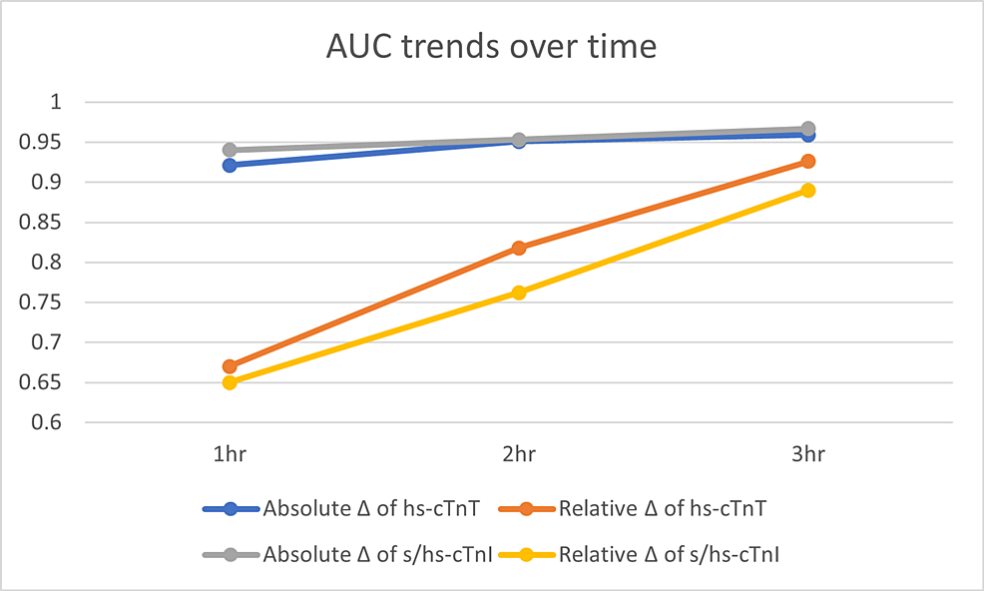
AUC trends over time Image credit: Authors of this study.

The AUC values in Table 14 are used to plot the AUC trends over a time graph as shown in Figure 9.

Discussion

Cardiac troponin changes (Δ) on a serial assessment are integral to the rapid “rule-in” and “rule-out” algorithms like the 0 h/1 h algorithm (blood drawn at 0 h and 1 h) or the 0 h/2 h algorithm (blood drawn at 0 h and 2 h) recommended by the current European Society of Cardiology (ESC) guidelines [18]. High sensitivity-cardiac troponin assays (hs-cTn) have allowed for a considerable shortening of the interval to second cardiac troponin assessment to 1 h/2 h. This meta-analysis validates and reinforces the use of absolute changes over relative changes in the algorithms recommended by current ESC guidelines. We observed a statistically significant difference for all comparisons of AUC values of absolute versus relative Δ we performed. There was no statistically significant difference in comparing s/hs-cTnI vs. hs-cTnT using absolute or relative changes at any time interval.

Our findings show a trend of a more dramatic increase in AUC values over time for relative changes than absolute changes as shown in Figure 7. The difference between AUC values between absolute versus relative changes is widest at 1 h and gets closer at 3 h time interval. So, our findings suggest that future research investigating a potential 0 h/30min algorithm should use absolute changes and agree with studies like Yokoyama et al. [19].

When hs-cTn assays are unavailable and conventional cTn assays are used instead, which might necessitate a delayed second troponin assessment, the superiority of absolute over relative Δ needs to be established at that delayed time interval. The above question is out of the scope of this meta-analysis since we examined the performance of s/hs-cTnI and hs-cTnT assays.

Limitations

Lack of access to databases like Embase, Web of Science, Cochrane, and Scopus is potentially a significant limitation of this report. Out of 12 groups shown in Table 3, three groups had only one report, and five had only two reports. All three groups with only one report in them were regarding relative changes. We used the data from only one report in those groups to test for statistical significance difference between AUC values of absolute versus relative changes.

Furthermore, in groups with only two reports, we were unable to establish the robustness of the analysis because we were unable to perform sensitivity analysis. The above reason also limited our ability to explore the possible causes of heterogeneity. We pooled data from sensitive-cTnI and highly sensitive-cTnI assays together, a potential cause of heterogeneity in those groups.

## Conclusions

Our analysis found absolute changes to be superior to relative changes in both hs-cTnT and s/hs-cTnI at 1, 2, and 3 h in the diagnosis of MI. We observed a statistically significant difference in all comparisons of AUC values of absolute versus relative Δ we performed. There was no statistically significant difference in comparing s/hs-cTnI vs. hs-cTnT using absolute or relative changes at any time interval. Our findings suggest that future research investigating a potential 0 h/30 min algorithm should use absolute Δ over relative Δ.

## References

[1] (2022). The top 10 causes of death. https://www.who.int/news-room/fact-sheets/detail/the-top-10-causes-of-death.

[2] Thygesen K, Alpert JS, Jaffe AS, Chaitman BR, Bax JJ, Morrow DA, White HD (2018). Fourth universal definition of myocardial infarction (2018). J Am Coll Cardiol.

[3] Amsterdam EA, Wenger NK, Brindis RG (2014). 2014 AHA/ACC guideline for the management of patients with non-ST-elevation acute coronary syndromes: a report of the American College of Cardiology/American Heart Association Task Force on Practice Guidelines. J Am Coll Cardiol.

[4] Eggers KM, Oldgren J, Nordenskjöld A, Lindahl B (2004). Diagnostic value of serial measurement of cardiac markers in patients with chest pain: limited value of adding myoglobin to troponin I for exclusion of myocardial infarction. Am Heart J.

[5] Reichlin T, Twerenbold R, Reiter M (2012). Introduction of high-sensitivity troponin assays: impact on myocardial infarction incidence and prognosis. Am J Med.

[6] Bahrmann P, Christ M, Bahrmann A (2013). A 3-hour diagnostic algorithm for non-ST-elevation myocardial infarction using high-sensitivity cardiac troponin T in unselected older patients presenting to the emergency department. J Am Med Dir Assoc.

[7] Page MJ, McKenzie JE, Bossuyt PM (2021). The PRISMA 2020 statement: an updated guideline for reporting systematic reviews. J Clin Epidemiol.

[8] Whiting PF, Rutjes AW, Westwood ME (2011). QUADAS-2: a revised tool for the quality assessment of diagnostic accuracy studies. Ann Intern Med.

[9] (2022). Chapter 6: choosing effect measures and computing estimates of effect. https://training.cochrane.org/handbook/current/chapter-06#section-6-3-1.

[10] Aldous SJ, Richards AM, Cullen L, Than MP (2011). Early dynamic change in high-sensitivity cardiac troponin T in the investigation of acute myocardial infarction. Clin Chem.

[11] Cullen L, Parsonage WA, Greenslade J (2013). Delta troponin for the early diagnosis of AMI in emergency patients with chest pain. Int J Cardiol.

[12] Irfan A, Reichlin T, Twerenbold R (2013). Early diagnosis of myocardial infarction using absolute and relative changes in cardiac troponin concentrations. Am J Med.

[13] Kitamura M, Hata N, Takayama T (2013). High-sensitivity cardiac troponin T for earlier diagnosis of acute myocardial infarction in patients with initially negative troponin T test--comparison between cardiac markers. J Cardiol.

[14] Boeddinghaus J, Nestelberger T, Twerenbold R (2019). High-sensitivity cardiac troponin I assay for early diagnosis of acute myocardial infarction. Clin Chem.

[15] Simpson P, Tirimacco R, Cowley P, Siew M, Berry N, Tate J, Tideman P (2019). A comparison of cardiac troponin T delta change methods and the importance of the clinical context in the assessment of acute coronary syndrome. Ann Clin Biochem.

[16] Kim JW, Kim H, Yun YM, Lee KR, Kim HJ (2020). Absolute change in high-sensitivity cardiac troponin I at three hours after presentation is useful for diagnosing acute myocardial infarction in the emergency department. Ann Lab Med.

[17] Liu L, Consagra W, Cai X (2022). Sex-specific absolute delta thresholds for high-sensitivity cardiac troponin T. Clin Chem.

[18] Collet JP, Thiele H, Barbato E (2021). 2020 ESC guidelines for the management of acute coronary syndromes in patients presenting without persistent ST-segment elevation. Eur Heart J.

[19] Yokoyama H, Higuma T, Endo T (2018). "30-minute-delta" of high-sensitivity troponin I improves diagnostic performance in acute myocardial infarction. J Cardiol.

